# FGFC1 Exhibits Anti-Cancer Activity via Inhibiting NF-κB Signaling Pathway in *EGFR*-Mutant NSCLC Cells

**DOI:** 10.3390/md20010076

**Published:** 2022-01-17

**Authors:** Jingwen Feng, Songlin Li, Bing Zhang, Namin Duan, Rui Zhou, Shike Yan, Jeevithan Elango, Ning Liu, Wenhui Wu

**Affiliations:** 1 Department of Marine Bio-Pharmacology, College of Food Science and Technology, Shanghai Ocean University, Shanghai 201306, China; m190300804@st.shou.edu.cn (J.F.); m190300802@st.shou.edu.cn (B.Z.); m200300873@st.shou.edu.cn (N.D.); zr13162832380@163.com (R.Z.); m180300683@st.shou.edu.cn (S.Y.); srijeevithan@gmail.com (J.E.); 2 Research Centre of the Ministry of Agriculture and Rural Affairs on Environmental Ecology and Fish Nutrition, Shanghai Ocean University, Shanghai 201306, China; slli@shou.edu.cn; 3 Engineering Research Center of Aquatic Product Processing & Preservation, Shanghai Ocean University, Shanghai 201306, China

**Keywords:** FGFC1, NSCLC, *EGFR*-mutant, cell cycle arrest, apoptosis, NF-κB pathway

## Abstract

FGFC1, an active compound isolated from the culture of marine fungi *Stachybotrys longispora* FG216, elicits fibrinolytic, anti-oxidative, and anti-inflammatory activity. We have previously reported that FGFC1 inhibited the proliferation, migration, and invasion of the non-small cell lung cancer (NSCLC) cells in vitro. However, the precise mechanisms of FGFC1 on NSCLC and its anti-cancer activity in vivo remains unclear. Hence, this study was focused to investigate the effects and regulatory mechanisms of FGFC1 on two NSCLC cell lines, *EGFR*-mutant PC9 (ex19del) and *EGFR* wild-type H1299. Results suggested that FGFC1 significantly inhibited proliferation, colony formation, as well as triggered G0/G1 arrest and apoptosis of PC9 cells in a dose- and time-dependent manner, but no obvious inhibitory effects were observed in H1299 cells. Subsequently, transcriptome analysis revealed that FGFC1 significantly down-regulated 28 genes related to the NF-κB pathway, including IL-6, TNF-α, and ICAM-1 in the PC9 cells. We further confirmed that FGFC1 decreased the expression of protein p-IKKα/β, p-p65, p-IκB, IL-6, and TNF-α. Moreover, NF-κB inhibitor PDTC could strengthen the effects of FGFC1 on the expression of CDK4, Cyclin D1, cleaved-PARP-1, and cleaved-caspase-3 proteins, suggesting that the NF-κB pathway plays a major role in FGFC1-induced cell cycle arrest and apoptosis. Correspondingly, the nuclear translocation of p-p65 was also suppressed by FGFC1 in PC9 cells. Finally, the intraperitoneal injection of FGFC1 remarkably inhibited PC9 xenograft growth and decreased the expression of Ki-67, p-p65, IL-6, and TNF-α in tumors. Our results indicated that FGFC1 exerted anti-cancer activity in PC9 cells via inhibiting the NF-κB signaling pathway, providing a possibility for FGFC1 to be used as a lead compound for the treatment of NSCLC in the future.

## 1. Introduction

Lung carcinoma is one of the most prevalent types of cancer and exhibits the highest mortality rate among all cancer types globally [1]. Non-small-cell lung cancer (NSCLC) accounts for over 80% of all lung cancers, with the characteristics of a low cure rate and high mortality [2]. Epidermal growth factor receptor (*EGFR*) gene mutation is an important driving factor in non-small cell lung cancer (NSCLC) [3]. Common sensitizing mutations were detected in 79.8% of patients: 57.1% had exon 19 deletions [4]. In recent years, selective targeted EGFR tyrosine kinase inhibitors (TKIs) have been widely developed and used for the treatment of NSCLC in those who have an *EGFR* mutation [5]. However, most of them are still far from being used clinically owing to their poor selectivity or potential toxicity [6]. The post-operative recurrence rate of NSCLC is still very high, and the overall survival rate of NSCLC patients remains low [7]. The effectiveness of the available treatment options remains limited. Therefore, it is of great importance to develop new chemotherapeutic agents and treatment strategies. In recent years, investigators have focused on searching for new bioactive compounds from natural products and exploring their anti-cancer effect and underlying mechanism, which would undoubtedly provide valuable information on NSCLC treatment [8].

The cell cycle is a complex and strictly controlled process, consisting of different phases, which plays a key role in cell growth and proliferation [9]. The cell cycle consists of the G0/G1 phases, S phases, and G2/M phases [10]. The G0/G1 phase is characterized by the synthesis of mRNA and protein required for DNA replication [11]. Cell cycle disturbance is one of the important markers of cancer. Numerous proteins play a critical role in cell cycle progression from recent studies, such as cyclins and cyclin-dependent kinase (CDKs) complex [12]. There was increasing laboratory evidence showing that many anti-cancer drugs can inhibit tumor growth by inducing cell cycle arrest [13]. Therefore, arresting the tumor cell cycle can suppress cell proliferation and induce cell apoptosis [14]. Cell apoptosis is a kind of programmed cell death, which is related to tumor formation and development. Many kinds of mechanisms such as gene regulation and signal transduction were involved. Inducing cell apoptosis is an effective way of cancer treatment [15]. Caspase-3 is known as an important mediator of programmed cell death and cell apoptosis [16]. Activation of caspases during apoptosis results in the cleavage of critical cellular substrates, including poly ADP-ribose polymerase (PARP), leading to cell apoptosis [17,18]. Therefore, inducing cell apoptosis is the therapeutic mechanism for many anti-cancer drugs.

Nuclear factor-κB (NF-κB) is a family of critical transcription factors of the inflammatory pathway and plays a significant role in the progression of various cancers, such as lung, breast, liver, pancreatic, multiple types of lymphoma, etc [19,20]. NF-κB normally exists in the cytoplasm in an inactive form as it is bound to IκB. NF-κB activation is regulated by the IκB kinase (IKK) complex, which phosphorylates IκB, resulting in subsequent ubiquitination and proteasome-dependent degradation [21]. Next, released NF-κB translocates to the nucleus and facilitates the transcription of its target genes, e.g., interleukin-6 (IL-6), tumor necrosis factor-α (TNF-α), and intercellular cell adhesion molecule-1 (ICAM-1) [22,23]. NF-κB is a crucial factor that controls the expression of pro-inflammatory cytokines, pro-survival, anti-apoptotic, and cell cycle regulators [24]. In consideration of the significant role of NF-κB signaling in carcinogenesis and tumor progression, targeting NF-κB has gradually become a hotspot of cancer therapy research [25]. Tingting Long et al. reported that Polygonatum sibiricum polysaccharides play an anti-cancer effect through NF-κB signaling pathways in lung cancer [22]. Zengyang Pei et al. also found that low doses of selenium may protect the prostate from prostatitis-induced cancer by inhibiting nuclear translocation of the NF-κB [26]. Given the key role of NF-κB in the development of cancers, it has become an excellent target for cancer therapy.

**Figure 1:**
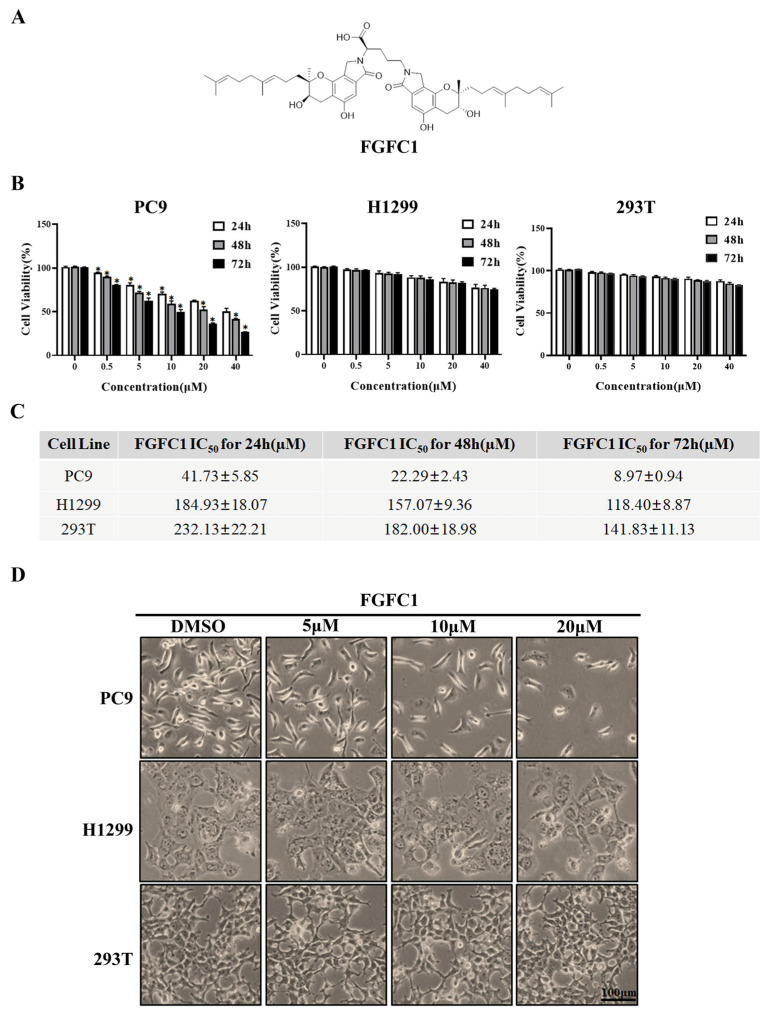

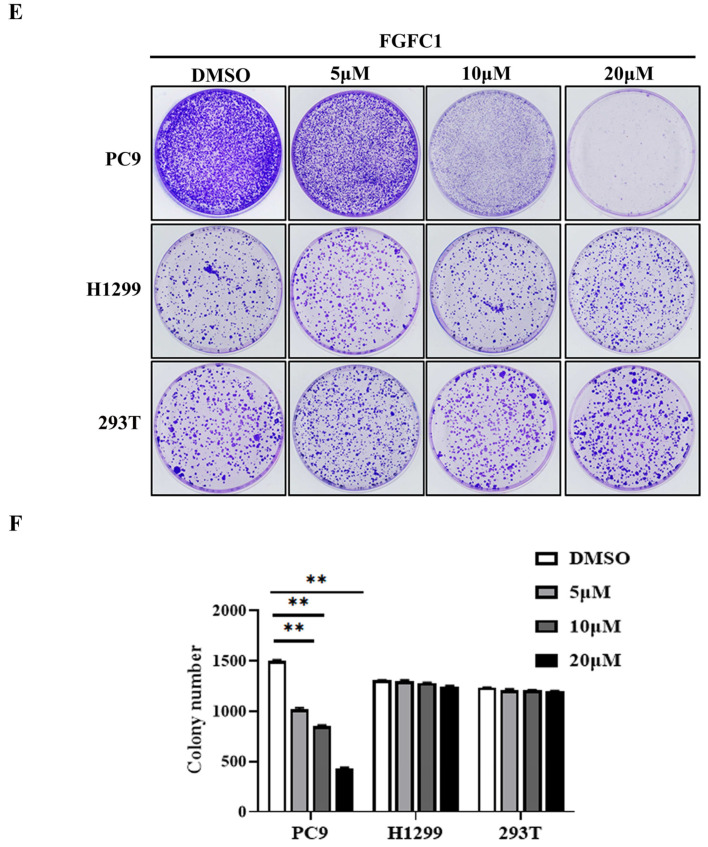
Effects of FGFC1 on the proliferation of NSCLC cells. (**A**) Chemical structure of FGFC1 (MW = 869.4947). (**B**) FGFC1 significantly inhibited the growth of NSCLC PC9 cells. PC9, H1299, and 293T cells were treated with increasing concentrations of FGFC1 (0, 0.5, 5, 10, 20, and 40 µM) for 24, 48, and 72 h, respectively. Cell viability was measured by the CCK8 assay and shown as relative viability compared to the untreated control. Each test was performed in triplicate. (**C**) The IC_50_ values of FGFC1 in PC9, H1299, and 293T cells were assessed and expressed as mean ± SD (*n* = 3). (**D**) The above-described cells were treated with different concentrations of FGFC1 (0, 5, 10, and 20 µM) for 72 h. Cell morphology was imaged under the microscope (200×). Scale bar = 100 μm. (**E**) FGFC1 dramatically suppressed colony formation of PC9 cells. NSCLC cells and 293T cells were treated with the indicated concentrations of FGFC1 (0, 5, 10, and 20 µM) for 10 days. Colonies were stained with 0.1% crystal violet and photographed. (**F**) Quantification of cell colony numbers in (**E**). All data were represented as the means ± SD for at least three independent experiments. (* *p* < 0.05 and ** *p* < 0.01 vs. the DMSO control).

In the past few decades, natural products have been considered as a potential and valuable source of anti-cancer drug candidates because of their multi-targeting potential and overcoming the disadvantages of monotherapy, such as side effects [27] and drug resistance. Fungi fibrinolytic compound 1 (FGFC1, 2,5-bis-[8-(4,8-dimethyl-nona-3,7-dienyl)-5,7-dihydroxy-8-methyl-3-keto-1,2,7,8-tertahydro-6H-pyran[a]isoindol-2-yl]-pentanoic acid, the chemical structure is shown in Figure 1A), is a rare compound with a molecular weight of 869 Da isolated from the culture of marine fungi *Stachybotrys longispora* FG216 (CCTCCM 2012272) [28]. FGFC1 has been proven to exhibit various biological activities such as thrombolytic, anti-inflammatory, and antioxidant activities [29]. Wu and others discovered that FGFC1 could enhance fibrinolysis, with no risk of bleeding through degrading fibrin protein in vitro and in vivo [30,31]. Keita Shibata et al. reported that FGFC1 effectively inhibited cerebrovascular inflammation by suppressing inflammatory cytokine mRNA expression [32]. Shike Yan et al. found that FGFC1 exerted an inhibitory effect on NSCLC PC9 cells in vitro [33]. However, the underlying mechanisms of FGFC1 on NSCLC and the anti-cancer effect in vivo deserve further investigation.

In this study, we evaluated the effects of FGFC1 on NSCLC cells both in vitro and in vivo and determined the inhibitory property of FGFC1 on the NF-κB pathway in NSCLC cells for the first time. Our results showed that FGFC1 possessed a significant anti-proliferative effect on *EGFR*-mutant PC9 cells via inducing cell cycle arrest and cell apoptosis through inhibition of the NF-κB signaling pathway. These results provide available information for understanding the anti-cancer effects and regulatory mechanism of FGFC1 on NSCLC, which will provide a sufficient base for FGFC1 to be promoted as a lead compound in NSCLC therapy.

## 2. Results

### 2.1. FGFC1 Significantly Inhibited PC9 Cells Viability In Vitro

To investigate the anti-cancer effects of FGFC1 on the NSCLC and the non-cancerous cells, dose- and time-dependent changes in PC9, H1299, and normal renal epithelial 293T cells viability were determined using the CCK8 assay after incubation periods of 24, 48, and 72 h. PC9 cells harbor *EGFR* mutation with exon 19 deletion, whereas H1299 cells carry wild-type *EGFR*. Here, H1299 cells were used as a control cell line to study the selective effect of FGFC1 on *EGFR*-mutant NSCLC [34,35]. Strikingly, treatment with FGFC1 caused a significant anti-proliferative effect on PC9 cells as indicated by dose- and time-dependent changes in cell viability, whereas a slight inhibitory effect only was observed in *EGFR* wild-type H1299 cells and 293T cells (Figure 1B). Figure 1C summarized the IC_50_ values of FGFC1 in these three cell lines. In PC9 cells, which were the most sensitive to FGFC1, the IC_50_ of FGFC1 after 24, 48, and 72 h of exposure were 41.73 ± 5.85, 22.29 ± 2.43, and 8.97 ± 0.94 µM, respectively. In H1299 and 293T cells, the IC_50_ was >100 µM after 72 h of exposure, suggesting that these cells were relatively less sensitive to FGFC1. As shown in Figure 1D, the observation under optical microscope confirmed that after a low concentration FGFC1 treatment, the pseudopodia of PC9 cells extended, part of cellular morphology turned into a spindle shape, resulting in significant changes in cell morphology and dose-dependent decrease in cell number. However, barely morphological changes were observed in H1299 cells and 293T cells, indicating that FGFC1 indeed exerted certain selectivity to PC9 cells with *EGFR* mutations. In addition, we performed a colony assay which is one of the indicators to evaluate the inhibitory effect of drugs on tumor cell proliferation. Compared with H1299 cells and 293T cells, FGFC1-treated PC9 cells reduced the number and size of the colonies in a dose-dependent manner. As shown in Figure 1F, after FGFC1 treatment, the number of cell colonies decreased from 1580 to 231, showing significant reductions in colony formation, further confirming the cell growth inhibition effect of FGFC1 on *EGFR*-mutant PC9 cells (Figure 1E,F).

### 2.2. FGFC1 Induced G0/G1-Phase Arrest and Apoptosis in PC9 Cells

**Figure 2:**
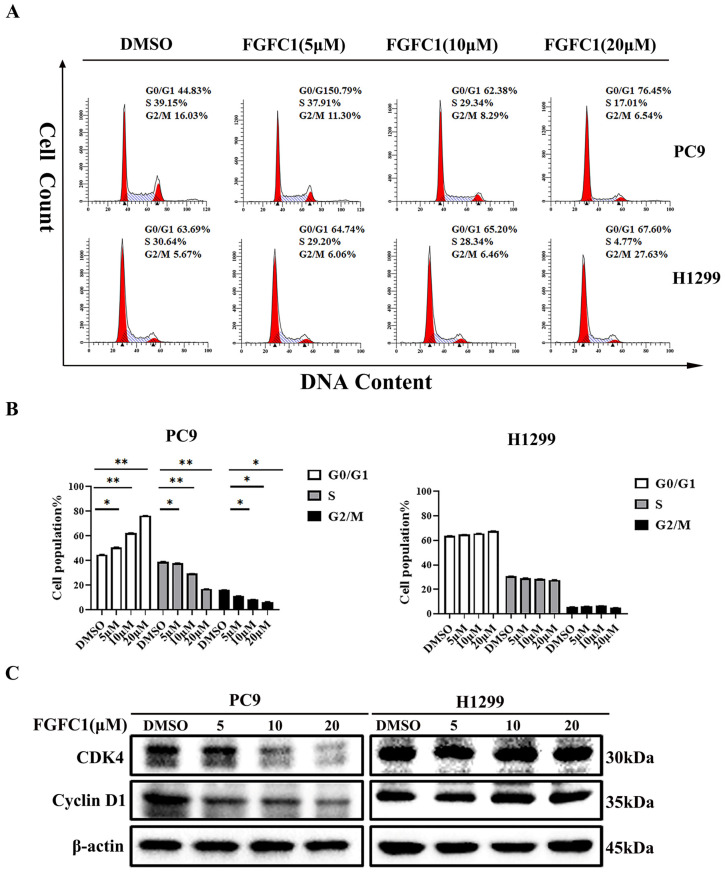

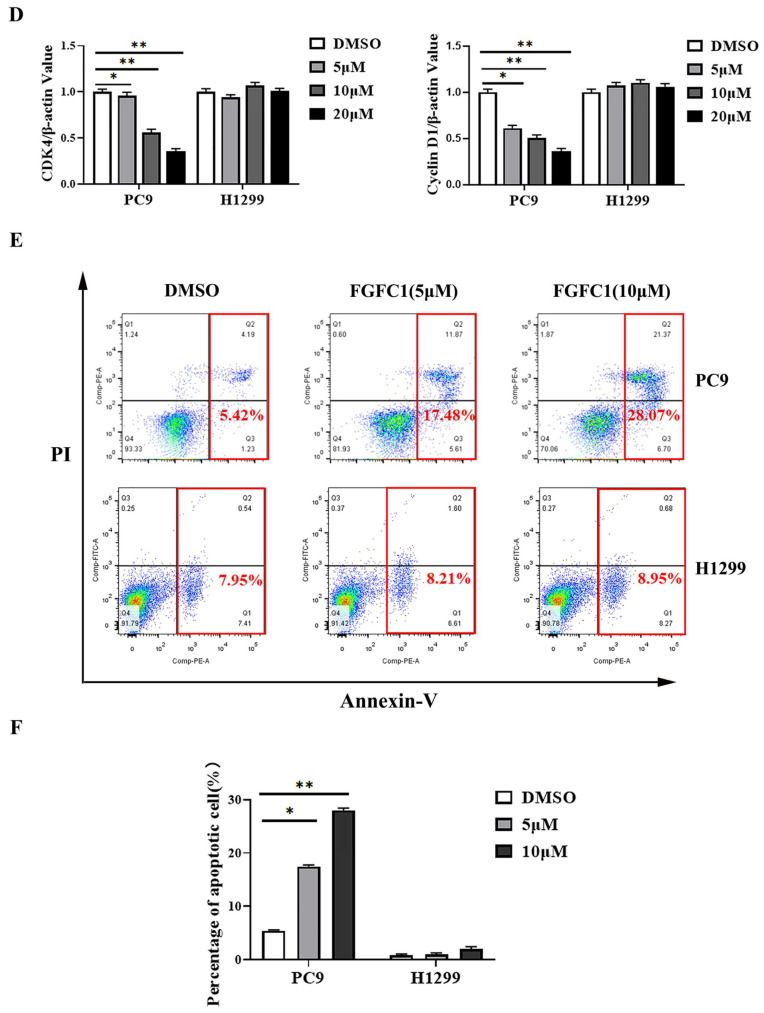

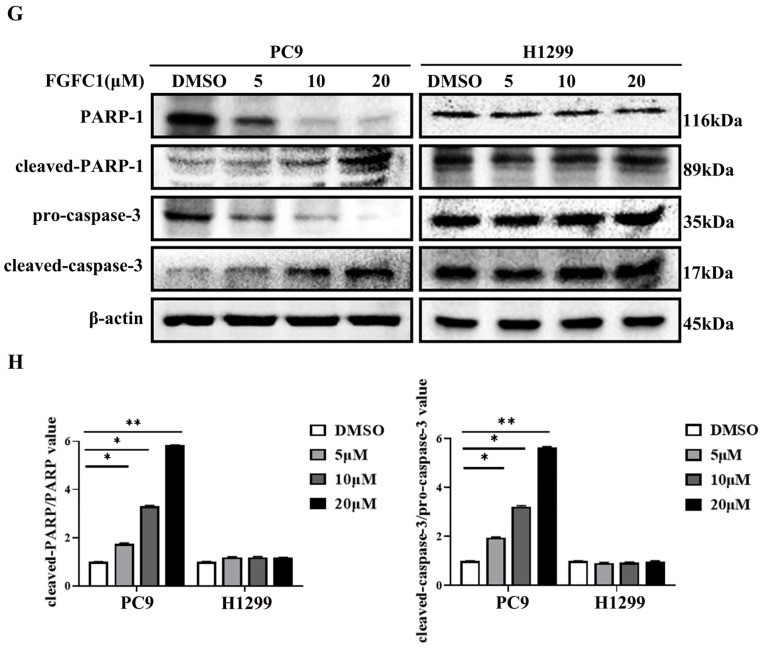
FGFC1 induced G0/G1-phase arrest and apoptosis in PC9 cells. (**A**) FGFC1 significantly increased G0/G1-phase accumulation in PC9 cells. PC9 and H1299 cells were treated with various concentrations of FGFC1 (0, 5, 10, and 20 µM) for 48 h, and the cell cycle distribution was analyzed by flow cytometry. The results were analyzed adopting the Modfit LT statistics program. (**B**) Data from three independent experiments were statistically analyzed and graphically depicted for PC9 and H1299 cells, respectively. (**C**) FGFC1 inhibited the expression of cell cycle-related proteins in PC9 cells. The above-described cells were treated with different concentrations of FGFC1 (0, 5, 10, and 20 µM) for 24 h. Protein expression of CDK4 and Cyclin D1 was detected by Western blotting. β-actin was used as an endogenous loading control. (**D**) Quantitative results of the protein levels in (**C**). CDK4/β-actin and Cyclin D1/β-actin ratios in Western blotting. (**E**) FGFC1 significantly enhanced PC9 cells death. PC9 and H1299 cells were incubated with the indicated concentrations of FGFC1 (0, 5, and 10 µM) for 48 h, and the cell apoptosis were detected by the Annexin-V FITC/PI staining assay. (**F**) The histograms indicated the percentage of total apoptosis. (**G**) FGFC1 increased the expression of apoptosis-related proteins in PC9 cells. PC9 and H1299 cells were treated with different concentrations of FGFC1 (0, 5, and 10 µM) for 24 h. PARP-1, cleaved-PARP-1, pro-caspase-3, and cleaved-caspase-3 protein levels were examined by Western blotting. β-actin was used as an endogenous loading control, accordingly. (**H**) Quantitative results of the protein levels in (**G**). cleaved-PARP-1/PARP-1 and cleaved-caspase-3/pro-caspase-3 ratios in Western blotting. All data were represented as the means ± SD for at least three independent experiments. (* *p* < 0.05 and ** *p* < 0.01 vs. the DMSO control).

To further determine whether or not the cytotoxic effect on NSCLC cells of FGFC1 is mediated by cell cycle arrest and/or apoptosis, PC9 and H1299 cells were treated with different doses of FGFC1 for 48 h. Subsequently, PI staining was used to analyze the distribution of cells at different stages of cell cycle by flow cytometry. The result demonstrated that following 0, 5, 10, and 20 µM FGFC1 treatment, the percentage of cells in the G0/G1 phase increased from 44.83% to 50.79%, 62.38, and 76.45% in PC9 cells, respectively. Nevertheless, limited effects of FGFC1 on the G0/G1 phase were observed in *EGFR* wild-type H1299 cells (Figure 2A,B). Given the effect of FGFC1 on the cell cycle, we further examined the expression of cell cycle-related proteins via Western blotting analysis. As expected, PC9 cells showed a dose-dependent decrease in CDK4 (0.95-fold, 0.56-fold, and 0.35-fold, respectively), and Cyclin D1 (0.61-fold, 0.50-fold, and 0.36-fold, respectively), after treatment with FGFC1 for 24 h compared with the DMSO-treated cells, but not in H1299 cells (Figure 2C,D). To further confirm the effects of FGFC1 on apoptosis in NSCLC cells, PC9 and H1299 cells were treated with different concentrations of FGFC1, and cell apoptosis was analyzed by flow cytometry. As presented in Figure 2E,F, a significant increase was observed in the percentage of PC9 apoptotic cells (5.42%, 17.48%, and 28.07% following 48 h treatment with FGFC1, respectively). Concomitantly, this was further evidenced by the increased level of cleaved-caspase- 3/pro-caspase-3 (1.96-fold, 3.22-fold, and 5.65-fold, respectively), and cleaved-PARP-1/PARP-1 (1.76-fold, 3.30-fold, and 5.80-fold, respectively), in FGFC1-treated PC9 cells (Figure 2G,H). In contrast, limited effects were observed in *EGFR* wild-type H1299 cells, which confirmed the selective inhibitory effects of FGFC1 on PC9 cells. These results suggested that FGFC1 exerted in vitro anti-proliferative effects on *EGFR*-mutant PC9 NSCLC cells through inducing G0/G1 cell cycle arrest and cell apoptosis.

### 2.3. FGFC1 Affected NF-κB Signaling Activation in PC9 Cells

**Figure 3:**
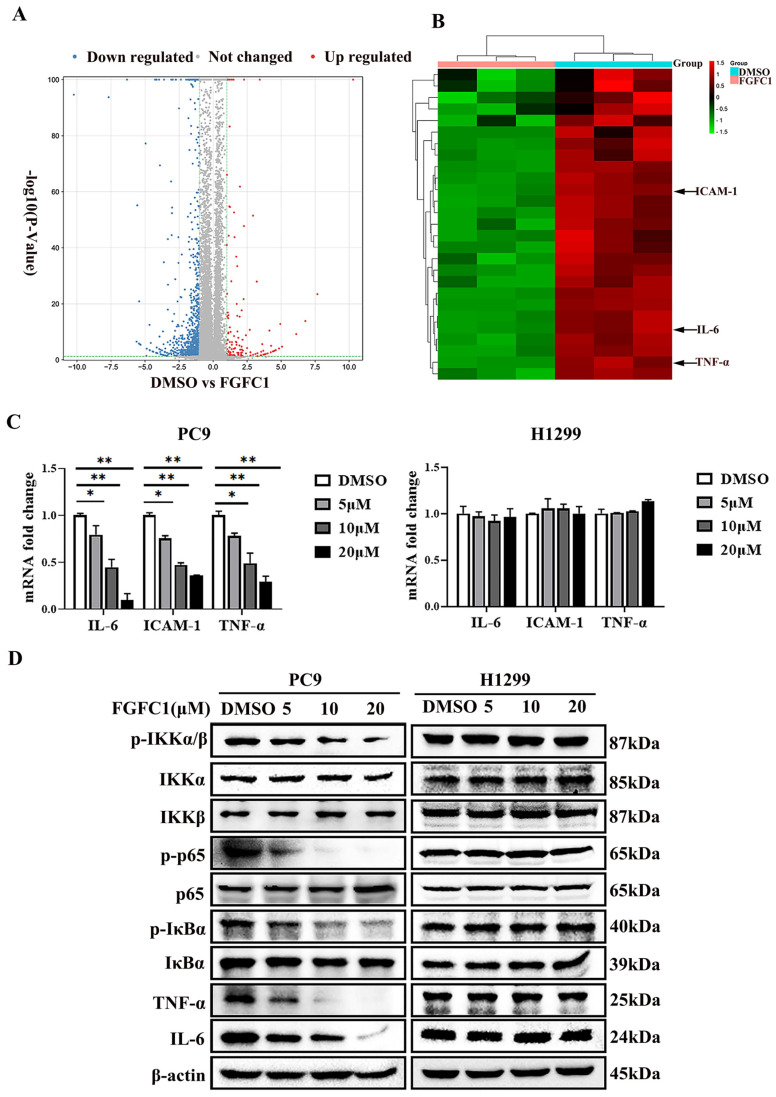

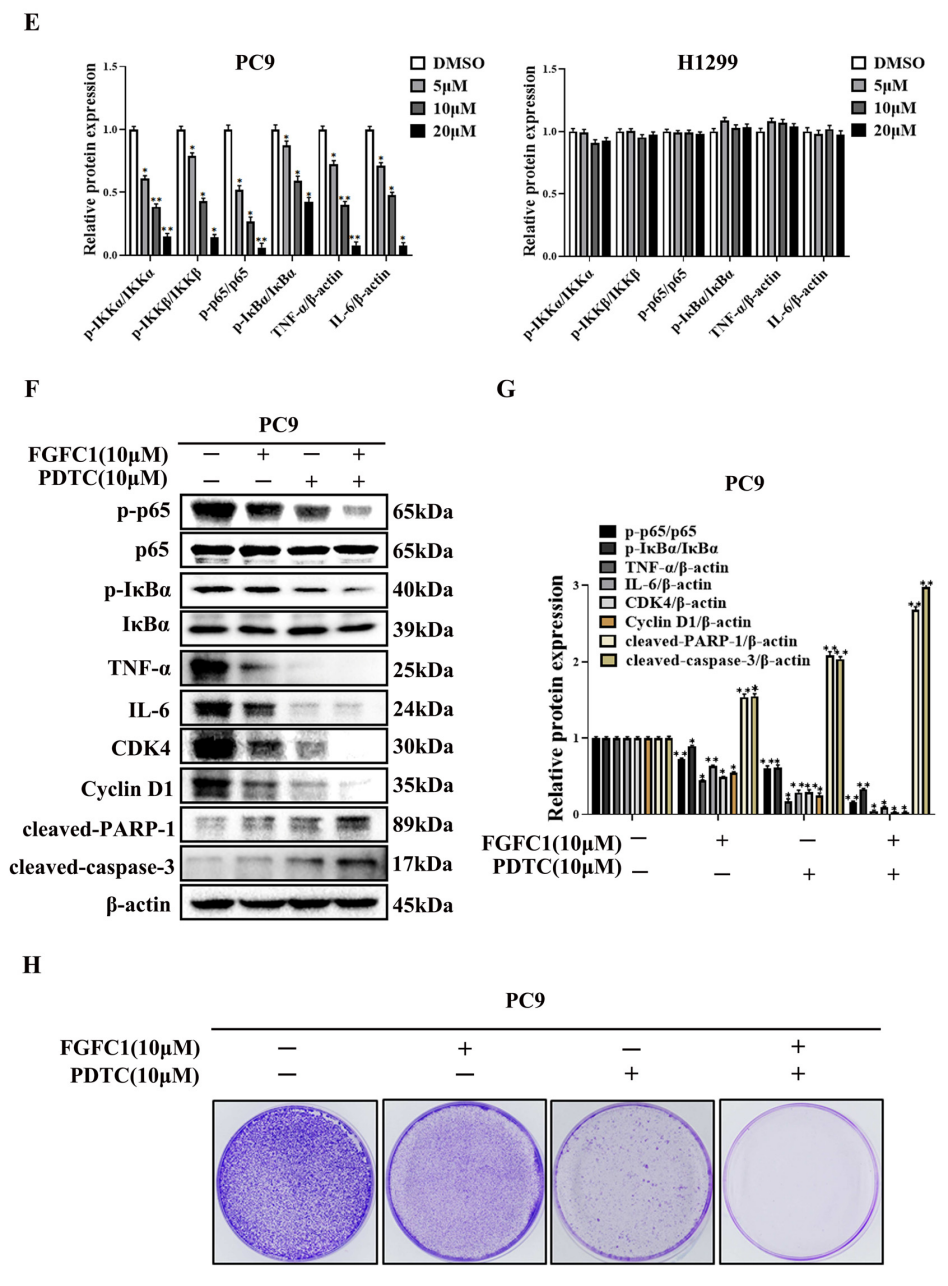

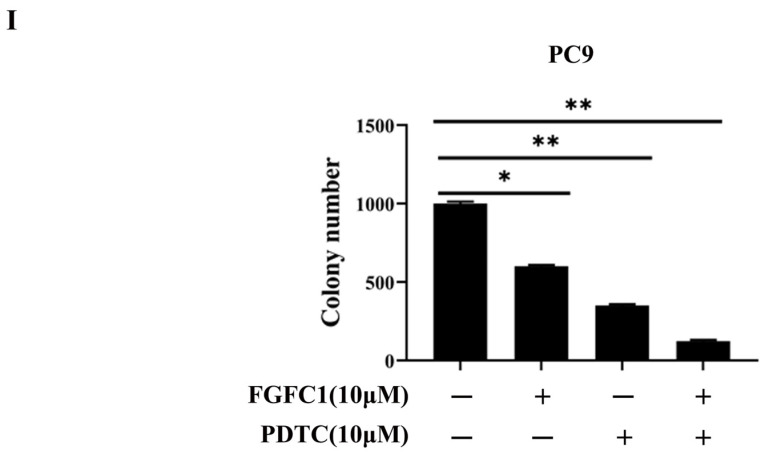
FGFC1 suppressed the NF-κB signaling pathway in NSCLC PC9 cells. (**A**) Volcano plot of DEGs, *x*-axis represents log2 transformed fold change, the *y*-axis represents -log10 transformed significance, red points represent up-regulated DEG, blue points represent down-regulated DEG, black points represent non-DEGs. (**B**) NF-κB-related genes were significantly suppressed by the treatment of FGFC1. PC9 cells were treated with DMSO or FGFC1 (10 µM) for 24 h, respectively. Total mRNA of different groups was prepared, heatmap of 28 DEGs that were significantly affected by FGFC1. Red indicated up-regulated genes and green indicated the down-regulated genes. Values were calculated from the regularized log transformation of fold change. The sequencing data were provided by The Beijing Genomics Institute (BGI). (**C**) Real-time PCR assays validated the inhibitory effects of the indicated FGFC1 (0, 5, 10, and 20 µM, 24 h) on NF-κB downstream genes (IL-6, ICAM-1, TNF-α). (**D**) FGFC1 remarkably suppressed the phosphorylation of the NF-κB pathway and inhibited its downstream targets. PC9 and H1299 cells were treated with the indicated concentrations of FGFC1 (0, 5, 10, and 20 µM) for 24 h. The expression of p-IKKα/β, IKKα, IKKβ, p-p65, p65, p-IκBα, IκBα, TNF-α, and IL-6 was examined by Western blotting. β-actin was used as an endogenous loading control. (**E**) Quantitative results of the protein levels in (**D**). p-IKKα/β/IKKα, p-IKKα/β/IKKβ, p-p65/p65, p-IκBα/IκBα, TNF-α/β-actin and IL-6/β-actin ratios in Western blotting. (**F**) PC9 cells were treated with FGFC1 (10 µM) and PDTC (10 µM) alone or in combination for 24 h. The expression of p-p65, p65, p-IκB, IκB, TNF-α, IL-6, CDK4, and Cyclin D1 was evaluated by Western blotting. β-actin was used as an endogenous loading control. (**G**) Quantitative results of the protein levels in (**F**). p-p65/p65, p-IκBα/IκBα, TNF-α/β-actin, IL-6/β-actin, CDK4/β-actin, Cyclin D1/β-actin, cleaved-PARP-1/β-actin, and cleaved-caspase-3/β-actin ratios in Western blotting. (**H**) FGFC1 significantly inhibited the colony formation of PC9 cells through the NF-κB signaling pathway. PC9 cells were treated with FGFC1 (10 µM) and PDTC (10 µM) for 10 days. Colonies were stained with 0.1% crystal violet and photographed. (**I**) Quantification of the cell colony numbers in (**H**). All data were represented as the means ± SD for at least three independent experiments. (* *p* < 0.05 and ** *p* < 0.01 vs. the DMSO control).

FGFC1 was proven to exert anti-proliferative effects on NSCLC PC9 cells. To further explore its anti-cancer mechanism in NSCLC, a high-throughput RNA sequencing (RNA-seq) was employed for the analysis of gene expressions in PC9 cells after DMSO and 10 μM FGFC1 treatment, respectively. The genes with *p*-value ≤ 0.05 and log2FC fold change ≥ 1 or ≤ −1 were selected as differentially expressed genes (DEGs). Gene expression analysis showed that 1167 genes were significantly differentially expressed between the FGFC1-treated group and the DMSO control group, including 1010 down-regulated and 157 up-regulated genes (Figure 3A). Strikingly, there were 28 significant down-regulated differential genes, which were NF-κB signaling pathway targets in FGFC1-treated PC9 cells, including the IL-6, TNF-α, and ICAM-1 (Figure 3B). To validate the RNA-seq results, we examined the expressions of IL-6, TNF-α, and ICAM-1 in FGFC1-treated PC9 and H1299 cells by qRT-PCR. As expected, compared with the DMSO control group, FGFC1 significantly reduced the transcription levels of IL-6 (0.79-fold, 0.45-fold, and 0.10-fold, respectively), ICAM-1 (0.76-fold, 0.47-fold, and 0.35-fold, respectively), and TNF-α (0.78-fold, 0.49-fold, and 0.29-fold, respectively), in a dose-dependent manner in PC9 cells; not in H1299 cells (Figure 3C). Accordingly, we further examined the effects of FGFC1 on NF-κB activity in NSCLC cells using Western blotting assay. As shown in Figure 3D,E, FGFC1 dose-dependently suppressed the phosphorylation of IKKα (0.61-fold, 0.38-fold, and 0.15-fold, respectively), IKKβ (0.79-fold, 0.43-fold, and 0.14-fold, respectively), p65 (0.52-fold, 0.27-fold, and 0.06-fold, respectively), and IκBα (0.87-fold, 0.59-fold, and 0.42-fold, respectively), in PC9 cells, while the levels of the total remained unaltered. Besides, FGFC1 also decreased the protein levels of TNF-α (0.72-fold, 0.40-fold, and 0.08-fold, respectively), and IL-6 (0.71-fold, 0.48-fold, and 0.08-fold, respectively), which are consistent with the results of qRT-PCR. Meanwhile, as a parallel control, we also examined the protein levels of these proteins in H1299 cells, but no evident inhibition was observed. To further explore the role of the NF-κB signaling pathway in the proliferation of NSCLC cells inhibited by FGFC1, we used pyrrolidine dithiocarbamate (PDTC) in combination with FGFC1 for the subsequent experiment. PDTC is a classical NF-κB inhibitor, which can suppress the phosphorylation of p65 [36]. PC9 cells were exposed to 10 µM FGFC1 and 10 µM PDTC alone or in combination for 24 h. The results clarified that co-treatment with FGFC1 and PDTC significantly suppressed the phosphorylation of p65 and IκBα, decreased the expression of NF-κB targets TNF-α and IL-6 compared with either drug alone group. Interestingly, co-treatment with FGFC1 and PDTC also had a significantly lower expression of CDK4 and Cyclin D1- critical proteins of the cell cycle than either drug alone group. Additionally, FGFC1 combined with PDTC enhanced the expression of apoptosis-related proteins, cleaved-PARP-1 and cleaved-caspase-3 in PC9 cells. Compared with the DMSO control group, the change fold of the above proteins in the FGFC1-treated group, PDTC-treated group, and FGFC1/PDTC-treated group is as follows: p-p65 (0.73-fold, 0.60-fold, and 0.16-fold); p-IκBα (0.89-fold, 0.62-fold, and 0.33-fold); TNF-α (0.44-fold, 0.17-fold, and 0.04-fold); IL-6 (0.63-fold, 0.29-fold, and 0.09-fold); CDK4 (0.49-fold, 0.29-fold, and 0.02-fold); Cyclin D1 (0.55-fold, 0.25-fold, and 0.03-fold); cleaved-PARP-1 (1.54-fold, 2.09-fold, and 2.68-fold); cleaved-caspase-3 (1.55-fold, 2.03-fold, and 2.98-fold). Therefore, these results demonstrated that FGFC1 could induce cell cycle arrest and cell apoptosis in PC9 cells through the NF-κB signaling pathway (Figure 3F,G). In addition, we performed a colony formation assay with the PDTC treatment experiment. It can be seen that compared with the DMSO control group and drug alone group, the colony formation of cells treated with PDTC and FGFC1 was greatly decreased. The result clarified that the number of cell colonies in the DMSO control group, FGFC1-treated group, PDTC-treated group, and PDTC/FGFC1-treated group were 1000, 600, 351, and 123, respectively (Figure 3H,I). Taken together, these results indicated that the NF-κB pathway was involved in the FGFC1-mediated anti-cancer process in NSCLC cells.

### 2.4. FGFC1 Inhibited NF-κB p-p65 Nuclear Translocation

**Figure 4:**
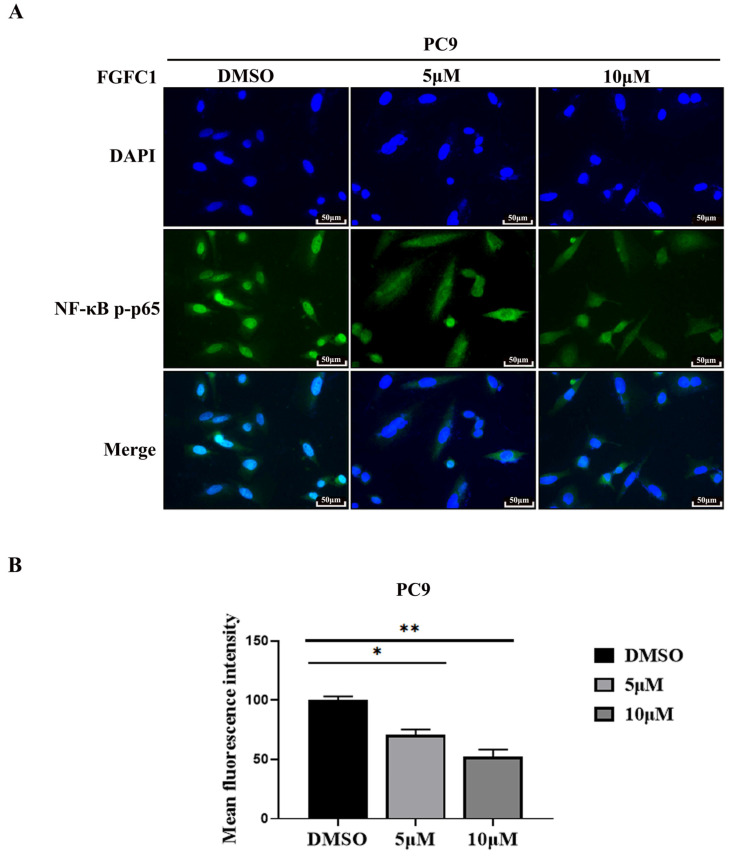
FGFC1 inhibited p-p65 nuclear translocation. (**A**) FGFC1 prevented p-p65 translocation into the nucleus. Shown are merged images (400× magnification) of DAPI staining and anti-rabbit Alexa488 recognizing primary p-p65 antibody (*n* = 3). (**B**) Quantitative analysis of the fluorescence intensity of p-p65 in (**A**). All data were represented as the means ± SD for at least three independent experiments. (* *p* < 0.05 and ** *p* < 0.01 vs. the DMSO control).

Nuclear translocation of activated transcription factor NF-κB commonly occurs in cancer and the inhibition of NF-κB has been proven to be an efficient therapeutic pathway for various cancers [37]. NF-κB activity is triggered with EGFR signaling in *EGFR*-driven NSCLC progression and NF-κB must translocate to the nucleus to regulate the target genes [38,39]. To investigate the effect of FGFC1 on p-p65 translocation in *EGFR*-mutant PC9 cells, we assessed the nuclear translocation of p-p65 using the immunofluorescence assay. The mean fluorescence intensity was 71 ± 4.26 and 52 ± 6.23, respectively, in different FGFC1-treated groups (Figure 4A,B).

### 2.5. FGFC1 Inhibited Tumor Growth In Vivo

**Figure 5:**
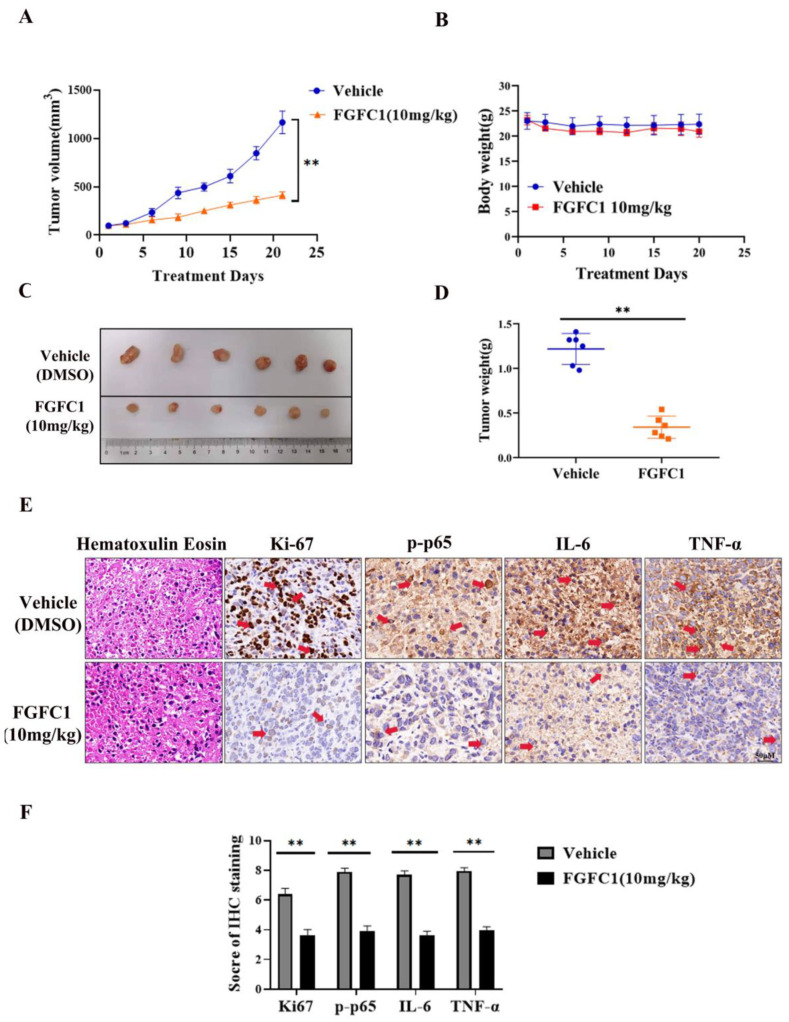
FGFC1 suppressed PC9 xenograft growth in immunodeficient mice. (**A**) Tumor volume was measured with a caliper from the beginning of the treatment by FGFC1 every three days. (**B**) When tumors reached a volume of approximately 100 mm^3^, mice were treated with vehicle (5% DMSO in PBS, ip) and FGFC1 (10 mg/kg, ip) once a day for 21 consecutive days. The body weight was quantified in each group. (**C**) Tumor pictures from control and FGFC1-treated mice. The PC9 xenograft tumors, which involved the vehicle (*n* = 6) and FGFC1 (10 mg/kg, *n* = 6) groups were photographed. (**D**) The scatter plot summarized the weight of the tumors. (**E**) FGFC1 decreased the expression of Ki67, p-p65, IL-6, and TNF-α in vivo. The expression of Ki67, p-p65, IL-6, and TNF-α in tumor tissues from nude mice was assessed by immunohistochemistry (400×), the red arrow indicates the positively stained nucleus (scale bar = 50 µm). (**F**) The IHC score of Ki67, p-p65, IL-6, and TNF-α was quantified by the IRS system (*n* = 10 fields of view). All data were represented as the means ± SD for at least three independent experiments. and ** *p* < 0.01 vs. the DMSO control).

Finally, we examined whether or not FGFC1 could prevent PC9 tumor xenograft progression in BALB/c athymic nude mice. The data showed that intraperitoneal injection of FGFC1 at 10 mg/kg efficiently suppressed tumor growth compared with vehicle treatment (Figure 5). The mean tumor volume increased from 98 ± 1.4 to 1166 ± 116.29 mm^3^ between days 1 and 21 in the vehicle control group, whereas the mean tumor volume increased from 98.5 ± 1.87 to 411.9 ± 36.33 mm^3^ in the FGFC1 group (Figure 5A). However, the body weight of mice did not decrease significantly (Figure 5B), suggesting that the toxicity of these therapies was tolerable. The average tumor weight in the FGFC1-treated group was lower than that in the control group (Figure 5C,D). In addition, the immunohistochemical analysis indicated that FGFC1 treatment remarkably reduced the expression of Ki-67 (0.56-fold), a marker for tumor cell proliferation [40], and significantly reduced the expression of p-p65, IL-6, and TNF-α (0.49-fold, 0.47-fold, and 0.50-fold, respectively), compared with the control group in tumor cells, which were consistent with the in vitro studies (Figure 5E,F). In conclusion, the results demonstrated that FGFC1 treatment significantly suppresses the growth of PC9 cells via regulating NF-κB signaling pathways, without a significant effect on the total body weight of the mice.

## 3. Discussion

As a marine-derived compound, FGFC1 consists of several bioactivities, including thrombolytic, anti-inflammatory, anti-oxidant, and anti-cancer activities [31,32,33]. The present study further investigated the anti-cancer effect of FGFC1 on NSCLC and its inhibitory effect on the NF-κB signaling pathway. The results showed that the semi-inhibitory concentration and colony formation abilities of the *EGFR*-mutant PC9 cells treated with FGFC1 were much lower than that of H1299. As known, cell cycle arrest and apoptosis are two effective mechanisms in the induction of cell death [41,42]. The cell cycle distribution diagram showed that FGFC1 arrested *EGFR*-mutated PC9 cells at the G0/G1 phase. Nevertheless, no remarkable effect was acquired in the *EGFR* wild-type H1299 cells. These results suggested that cell cycle arrest was one of the mechanisms in the growth inhibitory effect of FGFC1 against NSCLC. Cell cycle progression and division are regulated by checkpoint controls and sequential activation of cyclin-dependent kinases (CDKs), which are major targets for down-regulation in cancer [43]. To further explore the molecular mechanisms of the G0/G1 phase arrest induced by FGFC1, we examined the expression levels of cell cycle checkpoint proteins, Cyclin D1 and CDK4, which are essential for cell progression from G1 to the S phase for the start or G1/S checkpoint [44,45]. Our results showed that compared with H1299 cells, FGFC1 treatment significantly down-regulated the protein levels of CDK4 and Cyclin D1 in PC9 cells. In addition to cell cycle arrest, Annexin V and PI staining revealed that FGFC1 also induced cell apoptosis of PC9 cells. Consistent with flow cytometry results, FGFC1 treatment increased the protein levels of apoptosis-associated proteins cleaved-PARP-1 and cleaved-caspase-3 in PC9 cells. However, the same concentration of FGFC1 treatment could not induce cell cycle arrest and apoptosis in *EGFR* wild-type H1299 cells. As known, PC9 cells are defective in the *EGFR* gene with the exon19 deletion, but there is a wild-type *EGFR* in H1299 cells. These results indicated that FGFC1-induced cell death might be dependent on the *EGFR* status of NSCLC. However, the detailed mechanism needs to be further explored in the future.

NF-κB plays a well-known role in regulating immune response and inflammation, and growing evidence supports a major role in tumorigenesis [46]. Abnormal or constitutive activation of NF-κB p65 has been detected in many human malignancies [47]. Mingyue Lun et al. reported that the proteasome inhibitor Velcade is the first anti-cancer drug targeting NF-κB activation on the market. Velcade could inhibit the proliferation of breast cancer cells by suppressing the NF-κB pathway [48]. Nifedipine, known as a calcium channel blocker, had also been proved to inhibit the NF-κB pathway in lung cancer cells, as to explain the immunosuppressive effect of lung cancer [49]. Thus, NF-κB p65 is considered to be an interesting therapeutic target for cancer treatment [27]. To date, FGFC1-treatment has significantly decreased the expressions of activated NF-κB in transient middle cerebral artery occlusion (tMCAO) mice [50]. In addition, we have found that FGFC1 significantly down-regulated the expression of EGFR protein in PC9 cells [33]. However, the effect of FGFC1 on the NF-κB pathway in cancer cells is not clear. In the present study, RNA-seq results showed that FGFC1 down-regulated the expression of NF-κB target genes, including IL-6, TNF-α, and ICAM-1 in PC9 cells and qRT-PCR confirmed that FGFC1 indeed reduces the transcription levels of IL-6, TNF-α, and ICAM-1. Moreover, Western blot results revealed that FGFC1 can significantly decrease the phosphorylation of IKKα, IKKβ, p65, and IκBα and thus suppressed protein levels of NF-κB targets, IL-6 and TNF-α. Correspondingly, FGFC1 also inhibited the nuclear translocation of NF-κB p65 in PC9 cells. As expected, the activities of the NF-κB signaling pathway and the nuclear translocation of NF-κB p65 had not been affected by FGFC1 in *EGFR* wild-type H1299 cells. Furthermore, the PDTC and FGFC1 combined treatment group was found to have aggravated cell cycle arrest and apoptosis by enhancing the inhibitory effects of FGFC1 on the NF-κB pathway, finally inhibiting the colony formation of PC9 cells. The above results clarified that FGFC1 inhibited cell viability and induced cell arrest and apoptosis of *EGFR*-mutant NSCLC PC9 cells via negative regulation of the NF-κB signaling pathway. Additionally, as a crucial regulator of survival, cell cycle arrest, and apoptosis of cancer cells, the NF-κB signaling pathway has not only been linked to the oncogenic potential of the EGFR but is also involved in the acquisition of EGFR-TKIs resistance [39,51]. *EGFR* mutations are detected in approximately 30–65% of NSCLC patients in Asia [52]. Many studies illustrated that the EGFR pathway was crossed with the NF-κB-dependent pathway [53]. Mutations of *EGFR* triggers NF-κB activation via the proteasome-mediated degradation of IκBα. Meanwhile, transcriptional induction of NF-κB target genes exerted a positive feedback effect on EGFR signaling [54]. Activation of NF-κB and EGFR in a constitutive manner could be observed in multiple solid tumors and combined to provide oncogenic signals to cancer cells [53]. Recently, activation of NF-κB signaling has been proven to be vital for promoting resistance to EGFR-TKIs in cancers [55]. Masashi Fukuoka et al. revealed that the activation of NF-κB immediately after EGFR-TKIs treatment in PC9 cells acquired EGFR-TKI resistance [56]. Bivona et al. found that knocking down several components of the NF-κB pathway specifically enhanced EGFR-TKIs-induced cell death [57]. Galvani E et al. showed that a new type of irreversible EGFR-TKI CNX-2006 inhibited the cell proliferation via the NF-κB pathway, and was active against *EGFR*-mutated NSCLC models, both in vitro and in vivo [58]. In the subsequent study, whether FGFC1 can reverse the resistance-related mechanism of EGFR-TKI through the NF-κB signaling pathway, deserves further investigation. Our study would provide a sound rationale for future investigation in the clinical setting.

**Figure 6:**
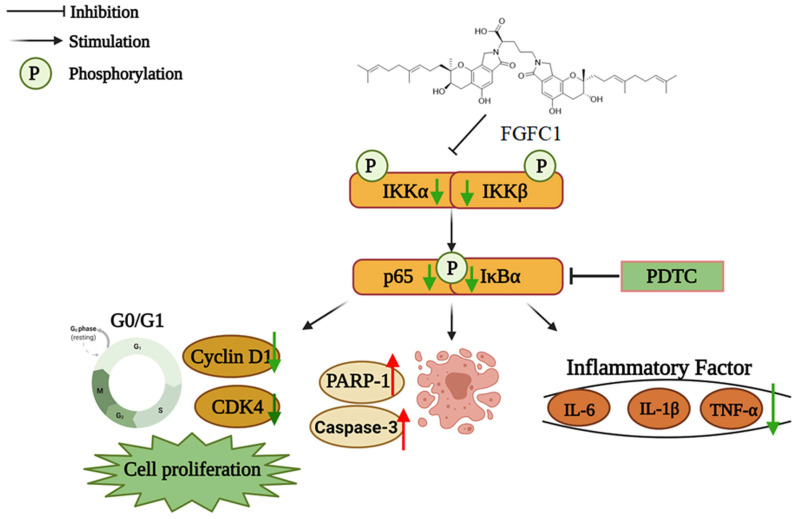
The proposed mechanism of the effect of FGFC1 on NSCLC cells. The solid arrow indicates direct stimulatory modification and the blocked arrow indicates direct inhibitory modification.

In vivo studies showed that FGFC1 was effective in reducing the tumor volume, size, and weight in a PC9 cell-xenograft mouse model. Compared with the control group, FGFC1 significantly inhibited tumor growth. However, mice body weight did not change following FGFC1 treatment. Additionally, immunohistochemical results showed that FGFC1 decreased the expression of Ki67, an indicator of cell proliferation in tumors [41], suggesting that FGFC1 effectively inhibited PC9 cells proliferation in vivo. To confirm whether FGFC1 exerts anti-cancer effects via the NF-κB signaling pathway in vivo, we analyzed the expression of p-p65, IL6, and TNF-α in PC9 xenograft tumors. Consistent with the in vitro results, the expression levels of these NF-κB pathway-related proteins expressions were also significantly decreased by FGFC1 (Figure 6).

## 4. Materials and Methods

### 4.1. Materials and Reagents

Fungi fibrinolytic compound 1 (FGFC1, purity > 98%) was extracted and purified from the methanol extracts of *Stachybotrys longispora*. FG216, as previously described [31]. Dantrolene dimethyl sulfoxide (DMSO) was purchased from Sigma Aldrich Co. (St Louis, MO, USA). Roswell Park Memorial Institute (RPMI)-1640 medium, Dulbecco’s Modified Eagle Medium (DMEM), phosphate-buffered saline (PBS) washing buffer, Fetal bovine serum (FBS), Trypsin-EDTA solution, and Penicillin-Streptomycin solution (PS) (100×) were all purchased from GIBCO (Carlsbad, CA, USA). Cell culture supplies were purchased from Costar (Corning, Inc., Cypress, CA, USA). Cell Counting Kit-8 (CCK8), RIPA lysis buffer, crystal violet, NF-κB inhibitor pyrrolidine dithiocarbamate (PDTC), and Cell Cycle and Apoptosis Analysis Kit were purchased from Beyotime Biotechnology Co. (Shanghai, China). Annexin V-FITC apoptosis detection kit was obtained from BD Biosciences (San Jose, CA, USA). TRIzol^®^ reagent was purchased from Invitrogen (Carlsbad, CA, USA). BCA Protein assay kit and RT Master Mix from Reverse Transcription kit, SYBR^®^ Green Real-time PCR Master Mix were acquired from TIANGEN (Shanghai, China). PVDF membranes were purchased from Millipore (Billerica, MA, USA).

The primary antibodies against β-actin, CyclinD1(92G2), CDK4 (D9G3E), PARP-1, cleaved-PARP-1, pro-caspase-3, cleaved-caspase-3, phospho-IKKα/β (Ser176/180)(16A6), IKKα, IKKβ (D30C6), phospho-NF-κBp65 (Ser536), NF-κBp65 (D14E12), phospho-IκBα (Ser32)(14D4), IκBα (L35A5), IL-6 (D3K2N), and TNF-α were obtained from Cell Signaling Technology (Beverly, MA, USA). Peroxidase-conjugated goat anti-rabbit and mouse secondary antibodies were obtained from Sigma Aldrich (St Louis, MO, USA).

### 4.2. Cell Lines and Cell Culture

The PC9 cell line (ATCC, CRL-32727, Manassas, VA, USA) and the H1299 cell line (ATCC, CRL-5803, Manassas, VA, USA) were from the American Type Culture Collection. PC9 cells contain *EGFR* exon 19 deletion mutation (E746-A750) [59], whereas H1299 cells possess the wild-type *EGFR* gene that can be used as control cells. These 2 cell lines were cultured in RPMI-1640. 293T normal renal epithelial cell lines were purchased from the Institute of American Type Culture Collection (ATCC, CRL-3216, Manassas, VA, USA) and cultured in DMEM. All culture media were supplemented with 10% FBS with 100 U/mL of penicillin and 100 μg/mL of streptomycin, and cells were cultured at 37 °C in 5% CO_2_. The cell lines were routinely tested to confirm that they were free of Mycoplasma.

### 4.3. Cell Proliferation Assay

PC9, H1299, and 293T cells were seeded into 96-well plates with 3 × 10^3^ cells/well in a culture medium and allowed to adhere overnight. The cells were then treated with various concentrations of FGFC1 (0, 0.5, 5, 10, 20, and 40 µM) for 24 h, 48 h, and 72 h, respectively, and 0.1% DMSO served as vehicle control. Each dosage was repeated in triplicate, and 3 independent experiments were performed. After treatment, 10 μL of CCK8 reagent was added to each well and incubated at 37 °C for 2 h. The medium was removed, followed by the DMSO dissolution. Finally, the absorbance of each well was measured at 450 nm by a Microplate Reader (BIO-TEK, Inc., Winooski, VT, USA). The concentration of FGFC1 required to inhibit cell proliferation by 50% (IC_50_) was calculated using GraphPad Prism 8.0 (San Diego, CA, USA) software for semi-log curve fitting with regression analysis.

### 4.4. Cell Morphology Observation

PC9, H1299, and 293T cells were seeded at 1 × 10^5^ cells/well into 24-well plates overnight and were treated with FGFC1 at different concentrations (0, 5, 10, and 20 µM) for 72 h, followed by morphology observation using an optical microscope (Nikon, Tokyo, Japan).

### 4.5. Colony Formation Assay

PC9, H1299, and 293T cells were seeded at approximately 400 cells per well in 6-well plates and were cultured with 4 mL of medium for 24 h. Then, the cells were treated with different concentrations (0, 5, 10, and 20 µM) of FGFC1 for 10 days, and 0.1% DMSO served as vehicle control. Next, cells were washed with PBS 2 times, fixed with 5 mL 4% paraformaldehyde for 15 min, and stained with 0.1% crystal violet for 15 min at room temperature. Finally, cell colonies were photographed under a microscope and the number of colonies in each well was counted for analysis.

### 4.6. Cell Cycle Analysis

The NSCLC cells were seeded in 6-well plates at a density of 2 × 10^5^ cells per well and were treated with FGFC1 (0, 5, 10, and 20 μM) for 48 h; DMSO served as vehicle control. Subsequently, cells were collected, washed with PBS, and fixed with 70% ice-cold ethanol at 4 °C overnight. After fixation, cells were washed with cold PBS and stained with 500 µL of propidium iodide (PI)/RNase staining buffer, followed by incubation on ice for 30 min. Cell cycle distribution was performed using FACSCalibur (Becton Dickinson, Franklin Lakes, NJ, USA). All experiments were performed in triplicate and yielded similar results. Obtained data were analyzed with Modfit LT 4.1 software (Verify Software House, Topsham, ME, USA).

### 4.7. Apoptosis Assay

Cell apoptosis was detected by flow cytometry using an Annexin V-FITC apoptosis detection kit. Briefly, NSCLC cells were seeded at 1 × 10^5^ cells/well into a 6-well plate overnight and were treated with FGFC1 at different concentrations (0, 5, and 10 µM) for 48 h. Subsequently, apoptotic cells were collected and then stained with Annexin V and PI in 1 × binding buffer at room temperature for 15 min. Finally, stained cells were analyzed using the FACSCelesta flow cytometer (BD Biosciences, San Jose, CA, USA) equipped with FlowJo V10 software (Flowjo, OH, USA).

### 4.8. Transcriptome Analysis

PC9 cells were selected for RNA-seq analysis using Illumina HiSeq 4000 (Illumina, San Diego, CA, USA). Briefly, after treatment with FGFC1, total RNA was extracted using Trizol reagent and was treated with mRNA enrichment method after it was purified by Oligo(dT)-attached magnetic beads. Purified mRNA was fragmented into small pieces, and the fragments were reverse transcribed and validated for quality control to get the final sequencing library. The library was sequenced on the BGISEQ-500 platform (BGI, Shenzhen, China). The sequencing data were provided by The Beijing Genomics Institute (BGI). The differentially expressed genes analysis was performed using the DESeq2 (v1.4.5) with *p*-value ≤ 0.05.

### 4.9. Quantitative RT-PCR (qRT-PCR)

After being treated with 0, 5, 10, and 20 µM FGFC1 for 24 h, NSCLC cells were collected for RNA extraction. RNA samples were then reverse transcribed into cDNA using the RT Master Mix from the Reverse Transcription kit. Quantitative analysis of target genes including IL-6, TNF-α, and ICAM-1 was conducted in triplicate using SYBR^®^ Green Real-time PCR Master Mix with 7500 Real-Time PCR System (Thermo Fisher Scientific, Invitrogen, Waltham, MA, USA). The relative expression of each targeted gene was calculated and normalized using the 2^−∆∆Ct^ method relative to reduced glyceraldehyde-phosphate dehydrogenase (GAPDH). The sequences of the primers used for qRT-PCR were as follows: IL-6 forward and reverse primers: 5′-GATGGATGCTTCCAATCTGGAT-3′ and 5′-AGTTCTCCATAGAGAACAACATA-3′; TNF-α forward and reverse primers: 5′-GCCAACGCCCTCCTGGCCAATG-3′ and 5′-CCCTTCTCCAGCTGGAAGAC-3′; ICAM-1 forward and reverse primers: 5′-ACCATGGAGCCAATTTCTC-3′ and 5′-ACAATCCCTCTCGTCCAG-3′, and the primers for the GADPH forward and reverse primers were 5′-CATATGGGGAAGGTGAAGGTCGGAGTC-3′ and 5′-GAATTCTTACTCCTTGGAGGCCATGTGG-3′. The GADPH primer was used as the negative control. Each assay was performed in triplicate.

### 4.10. Western Blotting Analysis

Cells were treated with different doses of FGFC1 (0, 5, 10, and 20 µM) or 10 µM PDTC for 24 h before being lysed with RIPA buffer on ice and quantified with a BCA assay kit. Equivalent amounts of proteins were resuspended in a loading buffer, boiled at 100 °C for 5 min, and separated by 10% or 12% sodium dodecyl sulfate-polyacrylamide gel electrophoresis (SDS-PAGE). Proteins were transferred to a 0.45 µM PVDF membrane. The membranes were blocked for 1 h at room temperature using 5% non-fat dry milk and subsequently incubated overnight at 4 °C with specific primary antibodies (1:1000). Then, the membranes were washed with TBST 3 times, followed by incubation with the secondary antibodies conjugated with horseradish peroxidase (HRP) (1:10,000) for 1 h. Protein bands were visualized with highly sensitive chemiluminescence (Bio-Rad, Hercules, CA, USA) and quantified using Image Lab software. β-actin were used as loading controls.

### 4.11. Immunofluorescence Detection

The expression of p-p65 in PC9 was determined by immunofluorescence staining. Cells were plated at 1.2 × 10^5^ cells/mL on glass coverslips in 6-well plates and treated with FGFC1 at different concentrations (0, 5, and 10 µM) for 24 h. After treatment, cells were washed with 1× PBS, fixed using 4% paraformaldehyde for 20 min, and then were permeabilized with 0.1% Triton X-100 in PBS containing 0.5% BSA (PBS-BSA) for 30 min. Subsequently, cells were blocked for 1 h using 1% BSA, incubated overnight at 4 °C with primary antibodies, p-p65. The slides were washed 3 times with PBS and stained for 30 min at room temperature with Goat anti-Rabbit IgG (1:500, Invitrogen, Waltham, MA, USA). Nuclei were counterstained with DAPI (4′,6-diamidino-2-phenylindole) (Invitrogen, Waltham, MA, USA) and fluorescence of the samples was captured using a confocal laser scanning microscope (A1þR, Nikon, Tokyo, Japan).

### 4.12. In Vivo Antitumor Experiment

BALB/c nude mice (male, 5–6 weeks old, 10~14 g) were obtained from the Jiesijie Experimental Animal Co., Ltd. (Shanghai, China), and were raised in a pathogen-free and temperature-controlled environment. PC9 cells (5 × 10^6^) were subcutaneously implanted into the right flank of the nude mice. Xenograft growth was monitored every other day using a digital caliber, and the tumor volumes (V) were measured using the following formula V = (L × W^2^) × 0.5, where L is the length and W is the width of the xenograft [53]. When tumors reached a volume of approximately 100 mm^3^, the mice were randomly divided into 2 groups of 6 mice each: vehicle control (5% DMSO, 3 times weekly, intraperitoneal (ip) injection) and FGFC1(10 mg/kg, 3 times weekly, ip injection). During the whole experimental period, the feed intake and motor activity of the mice were carefully observed, their body weights and tumor measurements were performed 3 times per week using a sterile Vernier caliper. At the end of the study (day 21), the mice were sacrificed, the tumors were excised carefully, and the tumor weights were measured. All animal experiments were by institutional animal care guidelines and protocols were approved by the institutional animal care and use committee (Permit Number: SHOU-DW-2018-054) of Shanghai Ocean University (Shanghai, China).

### 4.13. Immunohistochemistry

Immunohistochemical staining was performed by Shanghai RecordBio Co., Ltd. (Shanghai, China). Tumor sections were immunostained with specific anti-p-p65, anti-IL-6, anti-TNF-α, and anti-Ki67 antibodies. The images were captured using a Pannoramic MIDI scanner. The semiquantitative evaluation of immunohistochemistry with the expression of Ki67, p-p65, IL-6, and TNF-α through the immunoreactive score (IRS). The IRS system was applied to evaluate the immunoreactivity of each IHC marker [60]. Briefly, IRS = staining intensity × percentage of positive cells. Staining intensity was scored as 0 (negative), 1 (weak), 2 (moderate) and 3 (strong). Percentage of positive cells was scored as 0 (0–5%), 1 (6–25%), 2 (26–50%), 3 (51–75%) and 4 (76–100%). Ten visual fields from different areas of each specimen were chosen randomly for the IRS evaluation, and the average IRS was calculated as the final score.

### 4.14. Statistical Analysis

All experiments were repeated at least 3 times and the quantified data are expressed as the means ± SD unless otherwise indicated. Comparisons were made using one-way ANOVA analysis. *p*-value < 0.05 indicates statistical significance. Statistical analyses were performed with GraphPad Prism 8.0 software.

## 5. Conclusions

In conclusion, our results demonstrated that FGFC1 exhibited anti-cancer effects in *EGFR*-mutant NSCLC PC9 cells in vitro and in vivo via induction of G0/G1 cell cycle arrest and cell apoptosis mediated by the NF-κB signaling pathway. This study suggests that FGFC1 might be a promising lead compound that can be used as NSCLC treatment, therefore it merits further exploration.
